# Membrane Ultrastructure and T Cell Activation

**DOI:** 10.3389/fimmu.2018.02152

**Published:** 2018-09-25

**Authors:** Johannes Pettmann, Ana Mafalda Santos, Omer Dushek, Simon J. Davis

**Affiliations:** ^1^Sir William Dunn School of Pathology, University of Oxford, Oxford, United Kingdom; ^2^Radcliffe Department of Medicine, Medical Research Council Human Immunology Unit, Weatherall Institute of Molecular Medicine, University of Oxford, Oxford, United Kingdom

**Keywords:** T cell signaling, microvilli, invadosome-like protrusions, membrane topology, microscopy, microclusters, immunological synapse

## Abstract

The immune system serves as a crucial line of defense from infection and cancer, while also contributing to tissue homeostasis. Communication between immune cells is mediated by small soluble factors called cytokines, and also by direct cellular interactions. Cell-cell interactions are particularly important for T cell activation. T cells direct the adaptive immune response and therefore need to distinguish between self and foreign antigens. Even though decades have passed since the discovery of T cells, exactly why and how they are able to recognize and discriminate between antigens is still not fully understood. Early imaging of T cells was very successful in capturing the early stages of conjugate formation of T cells with antigen-presenting cells upon recognition of peptide-loaded major histocompatibility complexes by the T cell receptor (TCR). These studies lead to the discovery of a “supramolecular activation cluster” now known as the immunological synapse, followed by the identification of microclusters of TCRs formed upon receptor triggering, that eventually coalesce at the center of the synapse. New developments in light microscopy have since allowed attention to turn to the very earliest stages of T cell activation, and to resting cells, at high resolution. This includes single-molecule localization microscopy, which has been applied to the question of whether TCRs are pre-clustered on resting T cells, and lattice light-sheet microscopy that has enabled imaging of whole cells interacting with antigen-presenting cells. The utilization of lattice light-sheet microscopy has yielded important insights into structures called microvilli, which are small membrane protrusions on T cells that seem likely to have a large impact on T cell recognition and activation. Here we consider how imaging has shaped our thinking about T cell activation. We summarize recent findings obtained by applying more advanced microscopy techniques and discuss some of the limitations of these methods.

## Introduction

T cells are the central players in adaptive immunity. They control and orchestrate the immune response but are also involved in direct cytotoxicity toward tumors or virus-infected cells. A unique and crucial feature of T cells is their ability to distinguish between self and foreign peptides presented by major histocompatibility complex (MHC) proteins with high sensitivity and specificity. Antigen-presenting cells (APCs) process and present peptide-loaded MHC (pMHC) which is subsequently recognized by the antigen receptor expressed by T cells, i.e., the T cell receptor (TCR). Exactly how this binding event leads to receptor triggering, while self-peptides are ignored, is not well understood yet. Interestingly, T cell activation is known to be accompanied by profound changes in the spatial organization of TCRs and downstream signaling molecules.

T cells have been studied using numerous functional, genomic and imaging-based approaches. Microscopy has yielded especially valuable insights into the dynamics of T cell behavior and signaling *in vitro* and *in vivo*. Imaging membrane proteins on T cells during cellular activation led to the discovery of putative signaling assemblies, first in the form of the immune synapse, and later as precursor accumulations of TCRs, called microclusters (1–3). Recent technical developments in microscopy have allowed imaging of T cells in unprecedented temporal and spatial resolution. These developments have included single-molecule localization microscopy (SMLM), enabling super-resolution imaging, and lattice light-sheet (LLS) microscopy for 3D live, high-resolution cell imaging. Using these and other technologies, attention is now beginning to turn to the 3D topology of the cell membrane. Structures called microvilli or invadosome-like protrusions (ILPs) have been implicated in antigen probing and in the receptor triggering process (4–7). These structures are generally thought to be distinct from the accumulation of TCRs and other molecules in microclusters, observed on planar surfaces such as glass or supported lipid bilayers [SLBs; (1–3)].

In this review, we provide an overview of the common microscopy techniques used to image T cells (see Box 1) and discuss the types of membrane structures that have been observed in a variety of contexts. We consider the limitations in the imaging approaches used to characterize microclusters, ILPs and microvilli, and suggest that there could be substantial overlap in the cellular process resulting in the appearance of these structures in the course of imaging experiments.

Box 1 **Techniques used to study microclusters and membrane protrusions.**

The properties of the structures observed on the surface of T cells are to some extent dependent on the methodology used to study them. The substrate stimulating the cells can also have a significant influence on what structures are observed. However, there are also technical and physical limitations to resolving the full complexity, in particular the topology of the membrane. Here we give an overview of commonly used microscopy techniques used to study those phenomena.

**Confocal microscopy**

Conventional confocal microscopy allows high-contrast imaging at a diffraction-limited resolution of about 250 nm in the xy-plane. This technique was used when the immunological synapse was initially discovered by the group of Kupfer et al. between T cell-APC conjugates (8). The major drawback for such an application is the axial resolution (z direction) of only about 700 nm. Consequently, high-resolution images can only be obtained when the cells form a horizontal interface, as they would on glass.

**Total internal reflection fluorescence (TIRF) microscopy**

This diffraction-limited technique provides very high sensitivity, even allowing the tracking of single fluorescent molecules and their movement. By illuminating the sample from an angle, causing reflection of the light, only a 100–200 nm section of the sample next to the glass is illuminated, making this method highly suitable for imaging cell/transparent substrate interfaces. The original studies describing TCR microclusters utilized this method in combination with glass-supported bilayers for high-resolution live cell imaging (2, 3). Many subsequent studies used the same combination.

**Variable angle total internal reflection fluorescence (VA-TIRF) microscopy**

VA-TIRF is an adaptation of TIRF microscopy in which the angle of illumination is changed, allowing the mapping of the height of structures close to the glass. Jung et al. use this technique in combination with SMLM (see below) to map TCRs and other molecules in relation to the tips of microvilli (5).

**Single-molecule localization microscopy (SMLM)**

This super resolution microscopy technique allows the localization of fluorescently tagged molecules with a precision of less than 50 nm. Common implementations are photoactivated localization microscopy (PALM) and direct stochastic optical reconstruction microscopy (dSTORM). A combination of these methods was used by Razvag et al. to investigate the segregation of CD45 from the TCR (7). Currently, the number of colors in routine applications is limited to two. Furthermore, the acquisition can take a significant amount of time, making it mostly unsuitable for live cell imaging.

**Lattice light-sheet (LLS) microscopy**

Lattice light-sheet microscopy, as utilized by Cai et al. in their study of microvilli dynamics on T cells (6), is a microscopy technique that combines light sheet microscopy with structured illumination microscopy (SIM), a form of super-resolution microscopy. By producing a “sheet” of light for illumination, rather than relying on exclusion of out-of-focus light as in a confocal microscope, this method allows fast and gentle imaging. Compared to confocal microscopy, particularly the z resolution is significantly better, which allows, in combination with the outstanding scanning rate, the analysis of 3D structures like microvilli and their dynamics on live cells.

## The immunological synapse

During the late 1990s, enabled by new developments in confocal fluorescence imaging, Kupfer and Dustin and their colleagues described a large accumulation of TCRs at the center of contacts made by activated T cells with APCs or SLBs for the first time. The mature immunological synapse (IS), as it came to be called, consists of three subdomains: a central supramolecular activation cluster (cSMAC) containing TCRs, which is surrounded by a ring of ICAM-1 (Intercellular Adhesion Molecule 1) binding integrin LFA-1 (Lymphocyte function-associated antigen 1) molecules, called the peripheral SMAC (pSMAC), and a second outer ring called the distal SMAC (dSMAC), where the phosphatase CD45 accumulates. The exclusion of the large phosphatase CD45 from the TCR and its associated kinases has been proposed as a mechanism of T cell activation, referred to as the kinetic-segregation (KS) model (9). It might be expected that phosphatase exclusion to the dSMAC implicates the IS and KS-based signaling in T cell activation *per se*. However, early markers of signaling such as calcium fluxes precede the formation of the IS by minutes and the cSMAC has very little phosphotyrosine or downstream signaling effectors associated with it (3, 10). Furthermore, a synapse is not always observed at T cell/APC interfaces, depending on the APC used and the strength of the antigenic stimulus (11). Rather than being the driver of signaling during the earliest stages of T cell activation, the IS is more likely involved in processes like TCR downregulation (10, 12), via endocytosis (13) and the secretion of TCR-containing exosomes (14). Moreover, the IS is now considered to have a major role in the delivery of effector functions (15, 16) and co-stimulation (17).

## Microclusters

**Figure 1:**
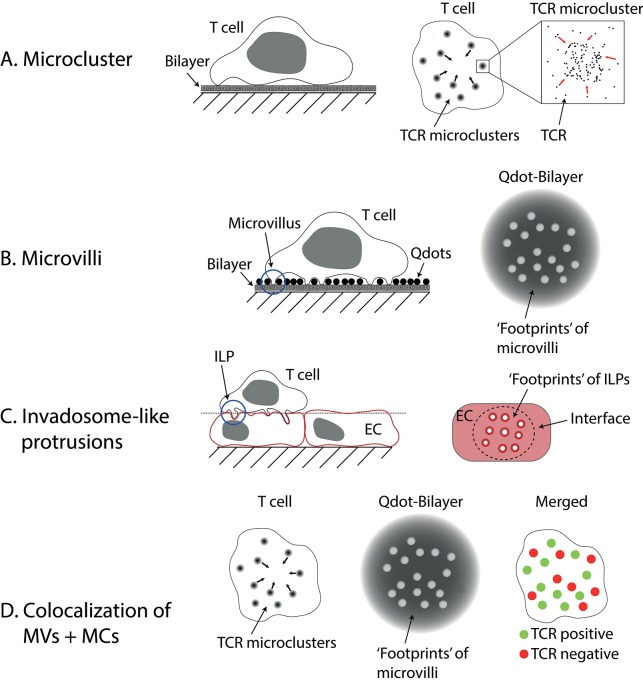
Microvilli and Invadosome-like protrusions. **(A)** Microclusters are accumulations of TCRs found on activated T cells imaged on lipid bilayers or glass. On bilayers, they can move centripetally to form the central supramolecular activation cluster (cSMAC). **(B)** Microvilli have been imaged using different techniques. Here a method called “quantum dot enabled surface contact mapping” is shown. T cells are placed on a bilayer coated with small (<20 nm), fluorescent quantum dots (Qdots). Microvilli can be observed in TIRF mode (see Box 1) by imaging the displacement of Qdots (“footprints”). **(C)** Invadosome-like protrusions (ILPs) are observed on T cells placed on a monolayer of endothelial cells (EC). When the membranes of the endothelial cells are imaged, “footprints” of ILPs can be seen as circular areas with decreased membrane dye fluorescence. The dotted line indicates the image plane shown in the right drawing. **(D)** Using a combination of the techniques described in **(A,B)** microclusters (MCs) and microvilli (MVs) can be imaged at the same time. Cai et al. (6) found that microclusters and microvilli colocalized on activated T cells. Notably, not all microvilli showed colocalization, however.

Total internal reflection fluorescence (TIRF)-based imaging (see Box 1) of the earliest stages of T cell activation prior to full IS formation has revealed the formation of TCR ‘microclusters’ (see Figure 1A). These submicron-scale structures form seconds after the T cell contacts an antigen-presenting surface. They are enriched in signaling molecules such as Lck, ZAP70, LAT, and SLP-76, and their formation precedes calcium fluxing (1–3, 18). After a cell has spread on SLBs that contain pMHC + ICAM-1, microclusters are observed to start moving toward the center of the contact to form the cSMAC (2, 3). Microclusters also form when T cells contact glass coated with anti-CD3 antibodies (1), but only mobile ligands (e.g., on SLBs or expressed by APCs) allow the movement of microclusters and the formation of a cSMAC. Within the cSMAC, the TCRs are mostly dissociated from downstream signaling molecules (3, 10).

Rather than arising in the IS at the cSMAC, continuous signaling appears to correlate with the formation of new microclusters in the p- and dSMAC (10), prompting the suggestion that these structures sustain T cell activation or are even the primary signaling units (19). However, other evidence from imaging cells during the earliest stages of activation is inconsistent with this view (see also Membrane Protrusions and Signaling). Inhibition of pMHC binding by a competitive antibody was shown to block the formation of new microclusters in the periphery, and also calcium fluxes, whereas existing microclusters and the cSMAC are mostly resistant to the treatment (10). A similar result can be obtained by using the actin polymerization inhibitor Latrunculin A (10). Actin is important for the formation of microclusters and, recently, actin foci that colocalize with microclusters have been identified (20). These Wiskott-Aldrich Syndrome protein (WASP)-dependent actin foci are synthesized *de novo* upon receptor triggering and contribute to downstream signaling. Interestingly, close inspection of the data of Yokosuka et al. also reveals that phosphorylated (*i.e*. activated) ZAP70 assembles into microclusters in the dSMAC early during T cell activation, without the detectable co-accumulation of TCRs (3, 10). These observations suggest that microclusters, like the synapse, might be the product of signaling rather than the cause of it.

## Microvilli

Lymphocytes and other leukocytes have a complex surface topology that is dominated to a large extent by round, finger-like protrusions termed microvilli (21). Scanning and transmission electron microscopy (SEM and TEM, respectively) has revealed that microvilli are 70–150 nm in diameter and from 100 nm to several μm in length (median length 300–400 nm), on resting T cells (5, 22). Microvilli can also be found on myeloid cells, but the surfaces of monocytes, polymorphonuclear leukocytes (22), and dendritic cells (23) are dominated by “ruffles”. Ruffles vary in size significantly, whereas the size of microvilli is less variable. Majstoravich et al. observed no large differences in the size of microvilli on primary human lymphocytes, primary murine lymphocytes and a pre-B lymphoma cell line, even though the median diameters of these cells vary significantly, suggesting that the function(s) of microvilli might depend on their size, which is therefore tightly regulated (22).

Until recently the microvilli of T cells had not been characterized dynamically and their actual functions are still unclear. Microvilli contain actin filaments and are highly mobile (6, 24). Since lymphocytes and most other cells are covered by a dense glycocalyx (25–27), which creates a barrier that acts against receptor/ligand interactions (28, 29), cells need to exert force to form close contacts, allowing ligand/receptor binding to occur. For T cells, microvilli have been implicated in force-driven penetration of the glycocalyx (4). Consequently, it seems reasonable to expect that the tips of the microvilli are the sites of initial TCR triggering.

Studies of membrane protrusions were previously hindered by the inability to image dynamic 3D structures, such as microvilli, with high resolution on live cells. The techniques used to study the IS and microclusters are generally not very suitable for such experiments (see Box 1). TIRF microscopy benefits from, but is also inherently restricted to imaging very close to a coverslip (within <200 nm). Confocal microscopy is generally capable of 3D imaging but suffers from significantly decreased resolution in the z-direction. Furthermore, scanning is too slow to capture a whole cell in high spatial and temporal resolution. Recognizing these limitations, Krummel and Betzig and their colleagues used a high-speed imaging technique with good z-resolution called lattice light-sheet (LLS; see Box 1) microscopy for imaging the movement of dynamic structures such as microvilli on live T cells (either in the resting state or forming APC conjugates), while using an adaptation of TIRF microscopy to visualize contacts formed at the tips of the microvilli [termed surface contact mapping (SCM); see Box 1 and Figure 1B; (6)] on SLBs. Microvilli observed using LLS imaging of live T cells interacting with DCs, or with SCM for T cells contacting SLBs, differed significantly in their height [see also Invadosome-like Protrusions]. Nevertheless, both approaches revealed microvilli moving rapidly over the entire interface in search of cognate antigen, surveying the majority of the opposing surface within a minute. Following encounter with cognate antigen, individual microvillar contacts were stabilized. Strikingly, microvillar search and stabilization were not decreased when ZAP70 was inhibited, implying that searching and stabilization are independent of downstream TCR signaling. When they imaged both the footprints of microvilli and the TCR using SCM, they observed strong colocalization (see Figure 1D). However, not every single microvillus was linked to the formation of a TCR microcluster (this might be susceptible to the threshold set to define TCR-positive structures), but microvilli lacking microclusters were selectively retained when cells were treated with Latrunculin A. Importantly, the authors found the microclusters that were localized on microvillar tips migrated centripetally, in the manner of “classical” microclusters (2, 3, 18). Together, these data suggest that structures previously observed in TIRF imaging experiments and referred to as “microclusters” might comprise, at least in some cases, the activation-dependent local accumulation of TCRs at the tips of microvilli.

## Invadosome-like protrusions

Invadosome-like protrusions (ILPs) are structures, initially identified using confocal microscopy, that form during the diapedesis of T cells (30). These membrane protrusions can penetrate deep into endothelial cells forming pores for subsequent transcellular migration. As shown by Sage et al., they are also involved in probing for antigens. In their study, endothelial monolayers were imaged after addition of T cells, and ‘footprints’ of the T cells pushing into the endothelial cells were observed [see Figure 1C; (4)]. Similar to microclusters and microvilli, the ILPs of activated T cells are enriched in TCRs, downstream signaling molecules and phosphotyrosine-containing molecules (4). In a small number of cells, structures resembling the cSMAC were found. Transmission electron microscopy revealed that ILPs have mean diameters of 350 nm in the absence, and 280 nm in the presence of antigen, respectively. Intriguingly, ILP tips are 9-fold more likely to form close contacts, that is sites of less than 20 nm intermembrane spacing very likely to facilitate TCR engagement of pMHC, than other regions of the contact. The authors speculated that microcontacts, small contacts observed to form when cells land on glass, are mechanically “frustrated” ILPs. One of the main differences between glass and cellular systems is the presence of a thick glycocalyx, and it is worth also noting that cells are many orders of magnitude less stiff compared to glass or plastic (31, 32). The stiffness of the substrate used has been shown to modify the response of T cells (33), which would, in principle, be explained by a force-dependent component of T cell triggering (34).

How ILPs might relate to microvilli has not been directly investigated. ILPs have mostly been characterized using endothelial cells as APCs, but they have also been found in B-T cell and DC-T cell conjugates (4), whereas MVs have mostly been studied on SLBs and in DC-T cell conjugates. Microvilli and ILPs share numerous similarities, including the enrichment of downstream signaling molecules, dependence of their formation on actin, and stabilization in the presence of cognate antigen (4–6). Curiously, the antigen-dependent stabilization of ILPs observed by Sage et al. was much more pronounced than that observed by Cai et al. for microvilli, implying that there could be functional differences among these structures. The long-lasting stabilization of ILPs might be specifically required for diapedesis, the process they were initially associated with. Notably, L-selectin, an adhesion receptor important for the initial binding required for diapedesis (tethering), is enriched on the tips of microvilli (5, 35–37). This localization to the tips is likely important, since redistribution of L-selectin using chimeric proteins impairs lymphocyte attachment under flow (35, 37).

Whereas the lengths of ILPs measured using TEM (mean length 430 nm) corresponds well with what has also been reported for microvilli using both TEM and SEM (median 300-400 nm), the diameters of these structures measured by electron microscopy are significantly different [~350 nm for ILPs vs. 70–150 nm for microvilli; (4, 5, 22)]. Cai et al. (6) reported much larger diameters for microvilli (mean ~540 nm) using both LLS and SCM microscopy, perhaps due to differences in cell activation status. When Cai et al. investigated the membrane topology of T cells interacting with SLBs, they observed only small variations in membrane/SLB separation across the contact (~50 nm), compared with the much longer microvilli seen in cell-cell conjugates or on resting cells (5, 22, 23). Similar topology was observed by Carbone et al. using scanning angle interference microscopy, in experiments in which giant unilamellar vesicles formed contacts with SLBs created by model (i.e., FKBP/FRB-based) receptor/ligand pairs in the presence of CD45 (38). We speculate that, to some extent, microvillus length is determined by the depth of the glycocalyx, which can be as much as 500 nm deep in the case of endothelial cells (27). The diameters of these structures might, however, be related to their role in interface scanning and antigen recognition (see Membrane Protrusions and Signaling). A striking observation consistent with this idea is that whereas human embryonic kidney cells do not form microvillar contacts with protein-coated glass surfaces in the manner of lymphocytes, they seem compelled to do so following their expression of a glycocalyx comprised of the membrane-anchored extracellular domain of CD45 (39). Perhaps the first task of these types of structures, therefore, is to punch through the glycocalyx (on both sides of a contact), allowing proper, cognate interactions. It is important to note, however, that in the context of natural killer and cytotoxic T cell interactions with their targets, marked membrane invaginations observed at the contacts are transient and that, in the course of minutes, the interfaces flatten and exhibit wider undulations (40). This suggests that the complex topology of the contacts is only important, if at all, during the earliest stages of interaction.

Based on these studies collectively, we propose that microvilli and ILPs are highly related structures whose assignment to either category depends only on how they are used by different types of cells: for probing antigen presenting cells for the presence of TCR ligands or, more vigorously, to initiate diapedesis. It is possible that the differences observed originate largely from the cell type used (murine/human, CD4^+^/CD8^+^, naïve/effector/memory) and the methods used to observe ILPs (mostly indirectly as membrane invaginations) and MVs (directly using light and electron microscopy). Future comparisons of structures observed using the same methods and cells would yield valuable insight into the variety and functions of membrane protrusions on T cells. Hereafter we use the term membrane protrusions to refer to both microvilli and ILPs.

## Membrane protrusions and signaling

A class of super-resolution techniques broadly named single-molecule localization microscopy, is based on the sequential excitation of small subsets of fluorophores, allowing the fluorescence point spread functions of diffraction-limited spots to be used to accurately determine the position of molecules with sub-diffraction resolution (see Box 1). Variable angle (VA)-TIRF microscopy (also see Box 1), on the other hand, allows measurement of the distance of fluorophores from a surface illuminated under TIRF conditions. Combining SMLM with VA-TIRF, Jung et al. characterized the distribution of the TCR and other molecules on the 3D surface of T cells (5). They observed that the TCR and L-selectin (a microvillus marker) were apparently enriched on resting T cells, i.e., pre-clustered on the tips of microvilli, and that CD44 formed rings around those sites. Latrunculin A completely blocked the formation of microvilli and clustering of the TCR (5).

Yi and Samelson (41) have suggested that membrane protrusions may create a structural scaffold for the formation of microclusters following T cell activation. In this way, they would serve as a physical barrier for the diffusion of molecules, enhancing signaling. The notion that membrane protrusions are dynamic, actin-containing foci also fits with the idea that force is an important contributor to T cell activation (34), and the response of the TCR/pMHC interaction to force seems in some cases to vary with the antigen (42). Lifetimes of agonist bonds are prolonged (due to formation of “catch” bonds) when forces are imposed on the interaction, whilst those of non-agonists are shortened (owing to “slip” bond formation); such effects were proposed to improve antigen discrimination (43, 44). Membrane protrusions might be ideally suited to divining such effects: first, the protrusion penetrates the glycocalyx, forming a close contact where interactions can occur, followed by a pulling force that elicits the catch/slip bond behavior of the interaction. It is unclear, however, why bond half-times would need to be extended in this way rather than through other, more straightforward thermodynamic processes, or how they especially would be selected for in the thymus. Also, it could be expected that adjacent adhesion molecules would have the effect of distributing and reducing local forces on the TCR, limiting such effects. Indeed, it is a strong argument against an important role for forces that adhesion molecules enhance TCR sensitivity rather than diminish it (45). It also needs to be emphasized that although forces have been detected using DNA-based nanoparticle tension sensors when T cells interact with immobilized anti-CD3 or pMHC (46), it is yet to be shown that this applies to T cell/APC contacts.

It was also proposed that membrane protrusions could add an important structural element to the kinetic-segregation model of phosphatase exclusion-based TCR signaling (41). The problem with this proposal is that although CD45 exclusion occurs upon ILP formation (4), the data for microvilli is somewhat equivocal. Chang et al. (39) observed spontaneous segregation of CD45 at microvillar-sized contacts formed by T cells interacting with artificial surfaces in a TIRF-based study and noted that this sufficed to initiate T cell activation. A similar study utilizing super-resolution imaging of T cells responding to glass-immobilized anti-CD3 antibodies reported a rather more complex reorganization of signaling proteins with a CD45-depletion zone ~250 nm in diameter, but without directly implicating membrane protrusions *per se* (7). Direct analyses of microvillar contacts analyzed using VA-TIRF and resting cells or SCM and activated cells, however, revealed only limited, if any, exclusion of CD45 (5, 6). One possible explanation for these discrepancies is that only ILPs, and the “frustrated” versions of these structures that may form on resilient artificial surfaces, may create compressive forces large enough to readily observe phosphatase exclusion. A smaller, less easily observed level of segregation, albeit one sufficient to initiate signaling, might only be achieved by more-subtle, microvillar-based cell-cell contacts. It is also possible that phosphatase exclusion occurs on length scales smaller than the resolution limit of TIRF microscopy. Further studies are needed to determine under what conditions, if at all, CD45 exclusion occurs at the tips of membrane protrusions during early cell-cell contact. This is presently very challenging, although the advent of single-molecule light-sheet imaging (47), or three-dimensional super-resolution imaging (48), offer ways to tackle this problem.

## Back to the beginning: the resting T cell surface

The remarkable, imaging-led progress in understanding the ultra-structural changes accompanying T cell activation has brought the field full circle to the problem of the resting, or “ground” state of the T cell, so that the drivers of signaling-dependent changes can be properly understood. The earliest electron microscopy-based data suggested that the TCR is pre-clustered on resting cells (49, 50). Subsequent single-molecule fluorescence-based studies of TCR stoichiometry and mobility implied instead, however, that the mobile TCRs expressed by T cells are largely if not wholly monovalent (51, 52), and that all TCRs are apparently mobile (53). The new proposal, i.e., that TCRs are freely diffusing and monovalent, was in turn quickly overtaken by new data obtained using SMLM, which supported the idea that the TCR was indeed pre-clustered in resting cells. Using high-speed photoactivated localization microscopy-based imaging, Lillemeier and colleagues proposed that the TCR is organized into “protein-islands” <70–140 nm in diameter (54). It was furthermore suggested that TCRs, LAT, CD4 and Lck were present in separate clusters on resting T cells on immobilized poly L-lysine, which then concatenate upon activation, yielding microclusters (54, 55).

But the notion that the TCR and other signaling proteins are pre-organized on resting cells has once again been challenged. Baumgart et al. (56) demonstrated that PALM and direct stochastic optical reconstruction microscopy (dSTORM) are generally prone to reporting artefactual protein clustering due to inhomogeneous stochastic fluorophore blinking, i.e., the erroneous detection of clusters due to overcounting. Whereas it was reported that the kinase Lck is clustered in domains with diameters of 50 nm (57), by titrating the levels of label, Baumgart showed that Lck is more likely homogeneously distributed in both resting and activated T cells. When Schütz and colleagues applied this approach to the TCR, they did not observe overt receptor clustering in non-activated CD4^+^ T cells in dSTORM and PALM experiments (58). An additional source of uncertainty is that by virtue of super-resolution experiments being TIRF-based, imaging has to be done on transparent, i.e., glass substrates that may or may not preserve the resting status of the imaged cell. Making matters worse, in many instances the cationic homopolymer poly L-lysine (PLL), widely thought to be inert, has been used to coat the glass surfaces used in the imaging experiments, presumably to enhance cell adherence. Santos et al. recently demonstrated, however, that PLL is not inert and that it produces levels of calcium signaling comparable to that measurable with the most potent combinations of activating antibodies in present use. Compared to cells in suspension, e.g., in hydrogels, Santos et al. showed also with super-resolution imaging that the organization of the TCR is profoundly altered following T cell contact with PLL-coated glass (47, 59). Most recently, using a variety of complementary non-invasive imaging/spectroscopy approaches, Huppa and colleagues showed that the TCRs that engage antigen are monomeric (60).

## Conclusion

Fluorescence-based light microscopy techniques, old and new, have already yielded paradigm-shifting insights into the ultrastructure and behavior of the T cell surface. Inevitably, controversies will arise as we gain experience with pioneering technologies and start to understand and accommodate their limitations. Because so much of what results in effective immunity occurs at the T cell surface, the stakes will always be high. For us, the two outstanding technological challenges are: (1) how do we study the resting T cell surface without perturbing it, and (2) how do we “get at” cell-cell contacts within seconds of the initiation of signaling, with the necessary time and spatial resolution. On the biology side, we would like to know: (1) what is the typical resting organization of a receptor expressed by a T cell, and what do exceptions to this behavior imply; or is the distribution of receptors and signaling proteins on the resting T cell surface best described as random? (2) What sub diffraction-limited ultrastructural changes accompany and, perhaps, drive early signaling, if any? (3) How and why do microclusters form, and how do they relate to microvilli, if at all? (4) Are membrane protrusions essentially all the same structures? (5) Why do T cells interrogate their targets using membrane protrusions, in any case? We can expect more surprises.

## Author contributions

All authors listed have made a substantial, direct and intellectual contribution to the work, and approved it for publication.

### Conflict of interest statement

The authors declare that the research was conducted in the absence of any commercial or financial relationships that could be construed as a potential conflict of interest.
